# Excavating the Missing Iron Age: Reinforcing Local Heritage & Identity in Sievi, Finland

**DOI:** 10.1007/s11759-023-09477-2

**Published:** 2023-04-19

**Authors:** Mirette Modarress Julin

**Affiliations:** Archaeology, University of Oulu, PL 1000, 90014 Oulu, Finland

**Keywords:** Public archaeology, Metal detecting, Archaeological heritage

## Abstract

In the small county in Northern Ostrobothnia, Finland, on a forest island amid fields, a few metal objects were found by metal detectorists. The finds suggested Iron Age dates and generated great interest, for both local people and researchers. As a result, an examination of the find locations was conducted by archaeologists, which was followed by excavations at one of the sites. In this article, under scrutiny is the interest excavations formulate in local contexts and how people relate to archaeological sites of their neighborhood. In addition, the role of metal-detecting in archaeology is considered. Generally, people in Finland are interested in the past of their home region. What about if there are only imperceptible remains and minor finds like fragments of ancient objects or shards of burned bone? Is material heritage important in everyday settings, and are people attracted to it? Here the aim is to relate some answers to these questions obtained by observations and discussions with the local people during the field research process.

## Résumé

Dans le petit comté de l'Ostrobothnie septentrionale en Finlande, sur un îlot forestier parmi les champs, quelques objets métalliques ont été trouvés par des détecteuristes de métal. Les éléments découverts suggèrent une datation à l'Âge de fer et ont généré le plus grand intérêt, tant de la part de la population locale que des chercheurs. Il en a résulté une étude des emplacements des objets trouvés sous la conduite d'archéologues, suivie de fouilles sur l'un des sites. Cet article se penche sur l'intérêt que les fouilles suscitent dans les contextes locaux et sur la manière dont les populations se sentent concernées par les sites archéologiques de l'espace environnant. Le rôle de la détection de métal dans l'archéologie est par ailleurs examiné. De manière générale, les populations en Finlande manifestent un intérêt pour le passé de leur région natale. Qu'en est-il s'il ne s'agit que de vestiges imperceptibles et de découvertes mineures comme des fragments d'objets anciens ou des éclats d'os carbonisé ? Le patrimoine matériel est-il important dans l'environnement quotidien, et les populations éprouvent-elles une attirance pour lui ? L'objectif est ici est de relier certaines des réponses à ces questions obtenues grâce aux observations et discussions avec les populations locales durant le processus de recherche sur le terrain.

## Resumen

En el pequeño condado en el norte de Ostrobotnia, Finlandia, en una isla forestal en medio de los campos, los detectores de metales encontraron algunos objetos metálicos. Los hallazgos sugirieron fechas de la Edad del Hierro y generaron un gran interés, tanto para la población local como para los investigadores. Como resultado, los arqueólogos realizaron un examen de las ubicaciones de los hallazgos, seguido de excavaciones en uno de los sitios. En este artículo, está bajo escrutinio el interés que provocan las excavaciones en los contextos locales y cómo las personas se relacionan con los sitios arqueológicos de su vecindario. Además, se considera el papel de la detección de metales en la arqueología. Generalmente, la gente en Finlandia está interesada en el pasado de su región de origen. ¿Qué pasa si solo hay restos imperceptibles y hallazgos menores como fragmentos de objetos antiguos o fragmentos de huesos quemados? ¿Es importante el patrimonio material en los escenarios cotidianos y las personas se sienten atraídas por el mismo? Aquí el objetivo es relacionar algunas respuestas a estas preguntas obtenidas por observaciones y discusiones con la población local durante el proceso de investigación de campo.

## Introduction

In this article, I examine local people’s relation to the archaeological heritage through a case study of the excavated site of Kiurunkangas, Sievi. Under scrutiny is the interest that excavations generate in local contexts and how people relate to imperceptible archaeological sites and minor finds of their neighborhood. Is material heritage important in everyday settings, and are people attracted to it and its investigation? The aim is to relate some answers to these questions obtained by observations, a short questionnaire, and discussions with people of the small community during the field research process. To set the local views on heritage in larger context parts of nationwide survey on cultural heritage is briefly considered.

**Figure 1:**
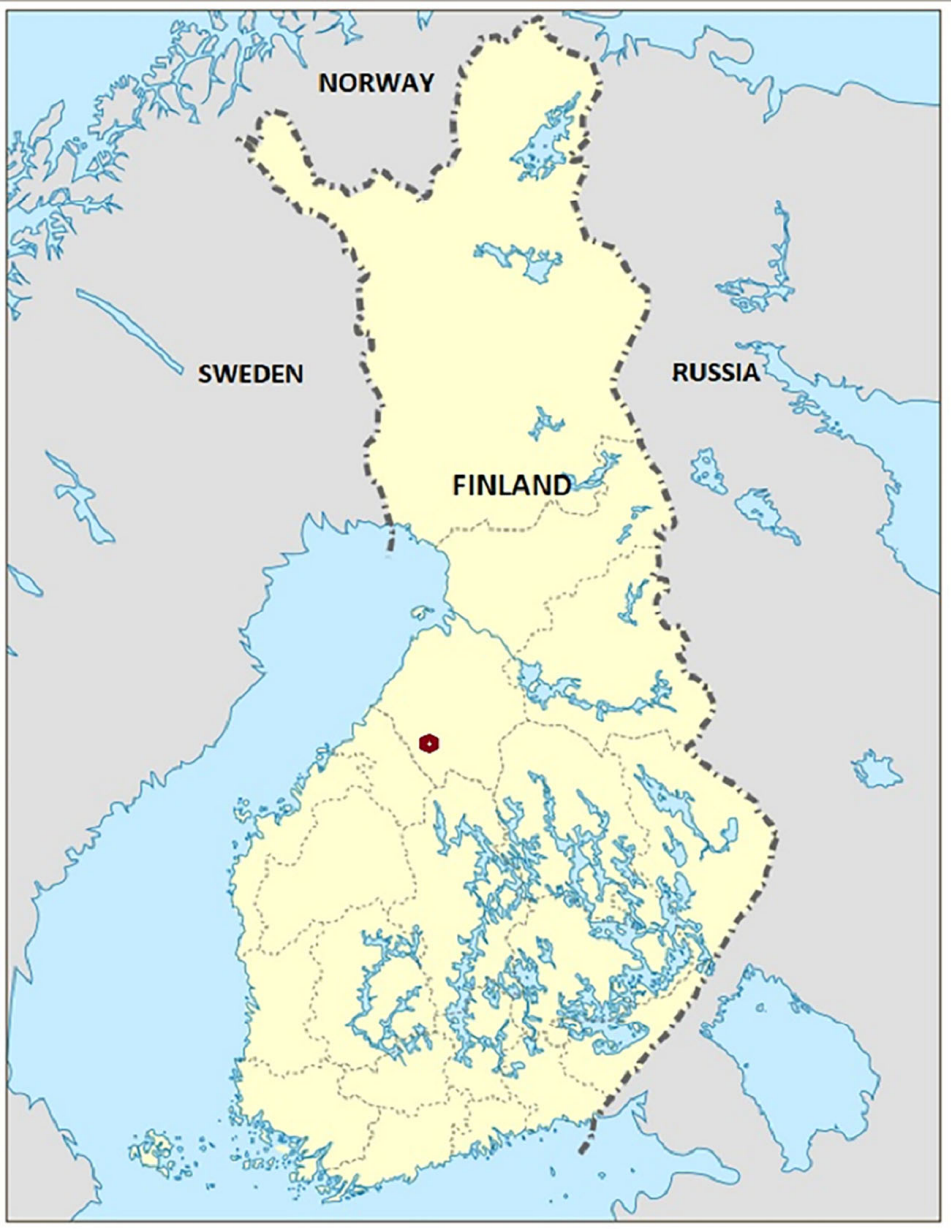
Map of Finland. Location of Sievi

The research in Kiurunkangas set off when a few metal objects were found by metal detectorists on a forest island amid fields in a small county of Sievi in Northern Ostrobothnia, Finland (Figure 1) in autumn 2018. The finds suggested Iron Age^1^ dates, and generated great interest, for both local people and researchers. As a result, an examination of the find locations was conducted by archaeologists from Oulu University, which was followed by survey of the area and excavations at one of the find sites in August 2019, 2020, and in autumn 2022.^2^ This also gave the possibility to inspect briefly the role of metal-detecting of archaeological sites, since the encounter with metal detector hobbyists had created some unease (Modarress et al., 2021).

Generally, people in Finland are interested in the past of their home region. The most visible remains such as prominent stone cairns, old churches or fortresses are usually considered as heritage, and a tourist attraction as well. Often, the sites of national or global heritage are highlighted. However, in a mundane context they could appear distant if the site mentioned is not situated in one’s own community or neighborhood. What I found intriguing when working on excavations at Kiurunkangas was my observation of the people’s attitude to indiscernible archaeological remains and minor finds.

## Public Participation

Though the excavation at Kiurunkangas was not planned as a community or public archaeology from the outset, it was clear that locals were to be involved one way or another, especially since the finds had roused a lot of interest. Accordingly,the project included public and/or community participation, depending on how these terms are defined (see e.g., Aalto, 2020; Gürsu, 2019; Kador, 2014; Matsuda & Okamura, 2011; Musteaţă, 2019; Richardson & Almansa-Sanchez, 2015; Shakour et al., 2019; Skeates et al., 2012; Smith & Waterton, 2009; Thomas, 2017).^3^ The most useful definition for public archaeology would be a broad one, as Matsuda (2019, p. 13) and also Grima (2019, p. 7) suggest and in this paper the concepts of community and public archaeology are overlapping. The community consists of many kinds of people from different origins, social backgrounds, age-groups, and the uniting factor does not need to be geographic either (e.g., Smith & Waterton, 2009). Nevertheless, since in this case ‘the public’ consists of various communities and people from Sievi and neighbouring regions, one of the main uniting factors is geographical, and people are mostly referred to with a term ‘local’, bearing in mind its multiple variations (see Meskell, 2005, p. 90).

There were some local volunteers and community participation both in the arrangements and in the field, though the agenda and managing were mainly done by archaeologists and the project included field-schools for archaeology students. In Finland, archaeological excavations must be led by an archaeologist with an MA or higher degree. Permission from the Heritage Agency is compulsory, and the director bears the responsibility for the various arrangements and the work on the excavation. Likewise, the written report containing all the information and data of the excavation process needs to be delivered to the Heritage Agency in due time after the excavations. Thus, excavations cannot be completely open for anyone to arrange or even to attend. The various stages of the field-project need personnel with acquired experience. Lay people often forget or do not even know that archaeology is much more than mere digging.^4^ The planning, the documentation, the lab work, and preparing the report take the best part of the research project. During the whole project, there should be time and effort also for analyzing, theorizing, and finally publishing the findings. In theoretical archaeological literature, it is common to highlight the importance and necessity of public participation, frequently from the social and ethical viewpoint. Theories are well formed and argued, however, the solutions would need more concreteness to be applied in the field.

In Finland, like many other countries public participation has become a trendy form of field-research during the last decade (e.g., Aalto, 2020; Äikäs & Itkonen, 2020; Mikkola, 2010; Moilanen et al., 2019; Ruohonen, 2019; Siltainsuu, 2012; Viljanmaa, 2015). Some excavation projects take a small participation fee from the volunteers, and some execute the public participation with the help of a grant. The precious and hard-to-get funding of excavations has become more easily attainable through public inclusion. One major grant allocated for public archaeology is *Dig it! archaeology initiative* -grant (Fi. *Mullankaivajat*, see the Finnish Cultural Foundation).^5^ The main requirement for attaining the grant is that schoolchildren and adult volunteers take part in the whole excavation process. The objective in public fieldwork is both pedagogical and to give a positive feeling of participation to those involved. Among other aims, excavations seek to raise awareness; and, as Matsuda and Okamura (2011, p. 9) state, the need to demonstrate the value of archaeology for the society in the market-led economy has become important. Likewise, Matsuda (2019, p. 17) also remarks that public archaeology resonates the dominant economic paradigm today.

## Traditional *Mutti-*porridge in the Market Square

**Figure 2:**
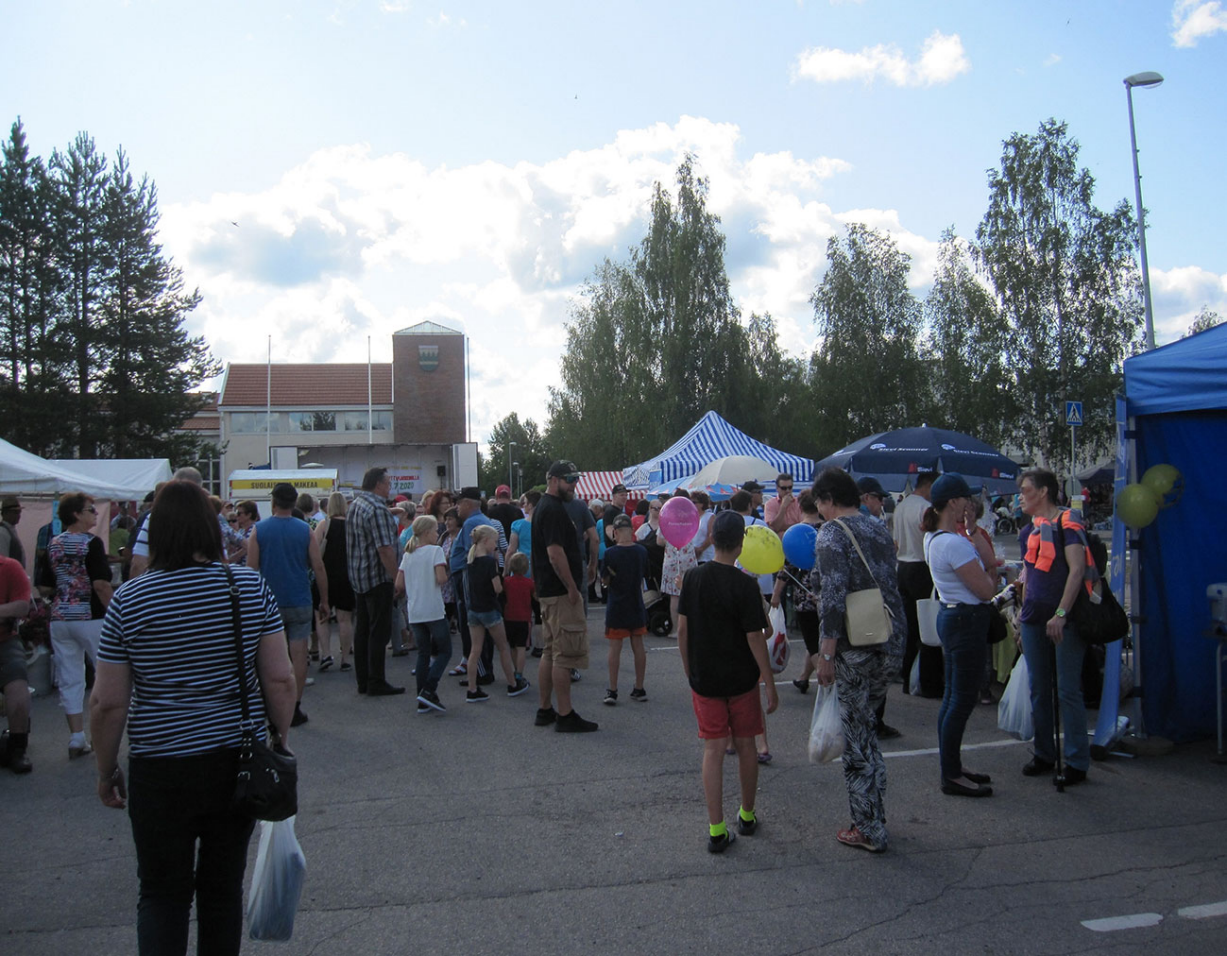
*Muttimarkkinat* fair in Sievi main square in July 2019. Municipality hall far back. Photograph: the author

Prior to the Kiurunkangas excavations, to inform local people of the coming research and to gain publicity for it, the author took part in an annual summer fair *Muttimarkkinat*^6^ (Figure 2), which was a big happening in the municipality of Sievi (obviously before the Covid 19 pandemic). On the market day people had a possibility to come to “meet an archaeologist” and chat about archaeology and heritage or show their finds and ask questions about them. Some came, and their conversations were not only connected to archaeological heritage, but much else as well. Also, three Stone Age finds (stone chisels), were brought for verification, and two persons came to talk about their discoveries of possible (new) sites which they hoped to be checked by an archaeologist. Conspicuously no metal finds were shown and not a single metal detectorist came, although they had been previously asking for identification of their finds from the local historian.

## Searching for Iron Age: Locality, Identity, and Archaeology

The find-spots of the Iron Age objects which started the research project in Kiurunkangas had been documented carelessly by the detectorists. The precise find spots were not known. Nevertheless, the shown/indicated site was chosen for more exact study, and the excavation in the end of August 2019 was a success in many ways, especially since Iron Age material was unearthed. (Hakamäki, 2020; Modarress et al., 2021) In general, in Finnish archaeological literature, the Sievi area and, more generally the regions in middle Northern Finland, have been considered “empty” of human habitation in the Iron Age period and only visited by occasional hunters (cf. Huurre, 1983, pp. 324–325). However, the reason for “emptiness” has been a research vacuum, since less archaeological research was done in Northern parts of Finland, which is less tensely populated region in Finland. Accordingly, recent studies are contradicting these ideas.^7^ The ancient local settlements are becoming more visible through archaeological record (Hakamäki, 2018; Hakamäki et al., 2022; Kuusela, 2013; Okkonen, 2013; see also Raninen & Wessman, 2015). After careful study the Kiurunkangas site turned out to be a multiperiod site with signs of activities from the Middle Stone Age to the late Iron Age (in Finland circa. 5000 BCE–1300 CE). Most excavation finds consisted of burned bone, quartz/stone tools, and various fragments of bronze and iron artefacts as well as some ceramic fragments in the 2020 and 2022) excavation. Uncovered structures consisted of a small fireplace and piled stones used probably for minor iron smelting or smithing. (See Hakamäki, 2020; Modarress Julin, 2021.)

**Figure 3:**
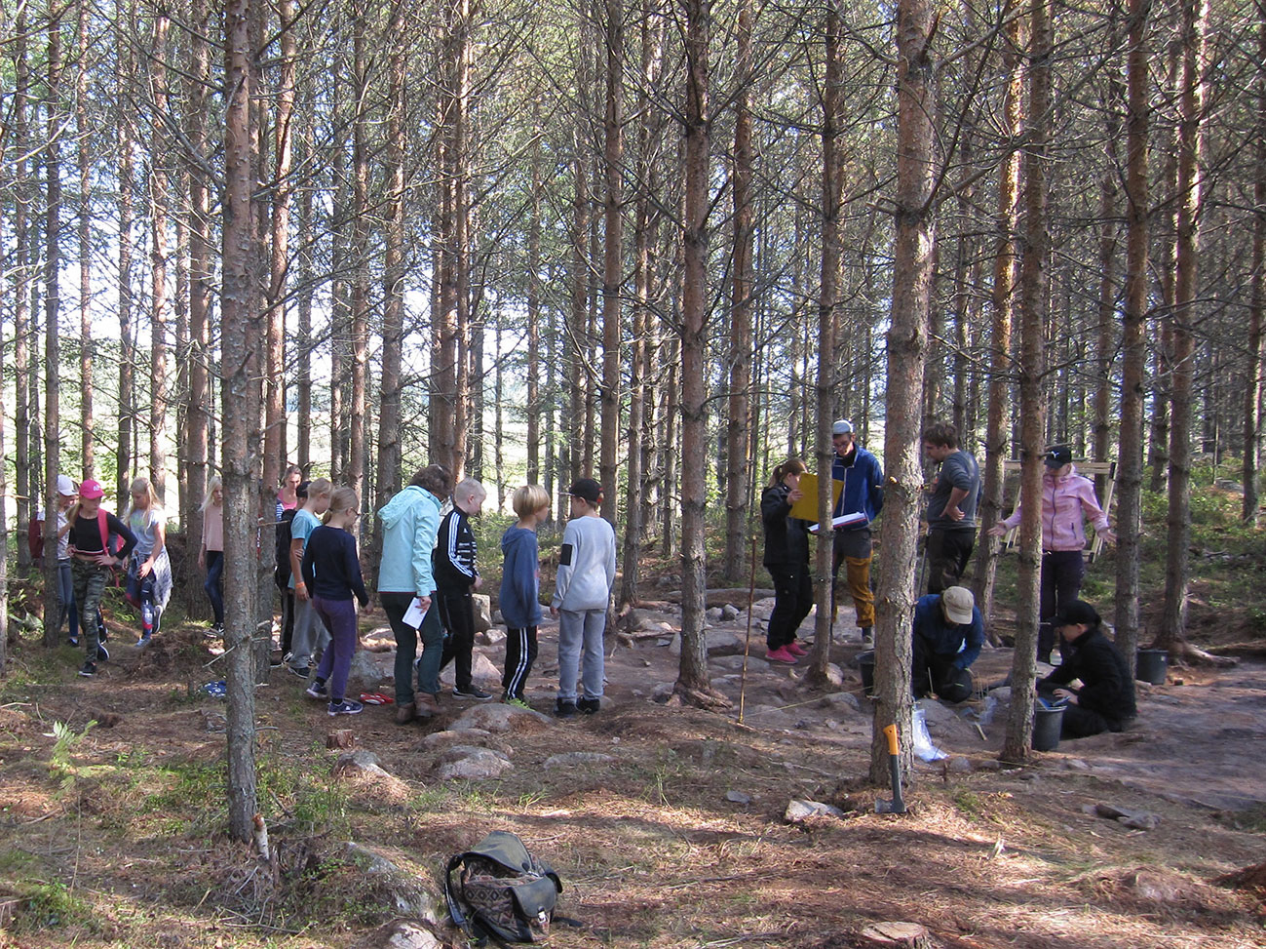
School children visiting the excavations in the summer 2019. Photograph: J. Okkonen

Another successful measure was involving the local school classes. Children came to visit the site with their teachers; almost each day of the excavations there were from one to three visits in groups of 15 to 25 students (Figure 3). An energetic and active local historian had mostly arranged these visits.^8^ After a tour in the excavations the students went to further discuss the topic of archaeology and heritage with their teachers at a nearby campfire site. They pondered upon what they had seen and how people had lived in the ancient times.

During the excavation period in 2019, I gave public lectures on archaeology and conducted a three-day course on excavating for 10 volunteers. In addition, “an archaeological walk” was arranged to familiarize participants with excavation sites and finds. These walks were extremely popular. About 30 to 40 persons attended, which was quite a good amount for the small county. Not only the excavation trench and the most spectacular finds were marveled at, but also the smallest bone or quartz finds were a matter of wonder to the participants.

**Figure 4:**
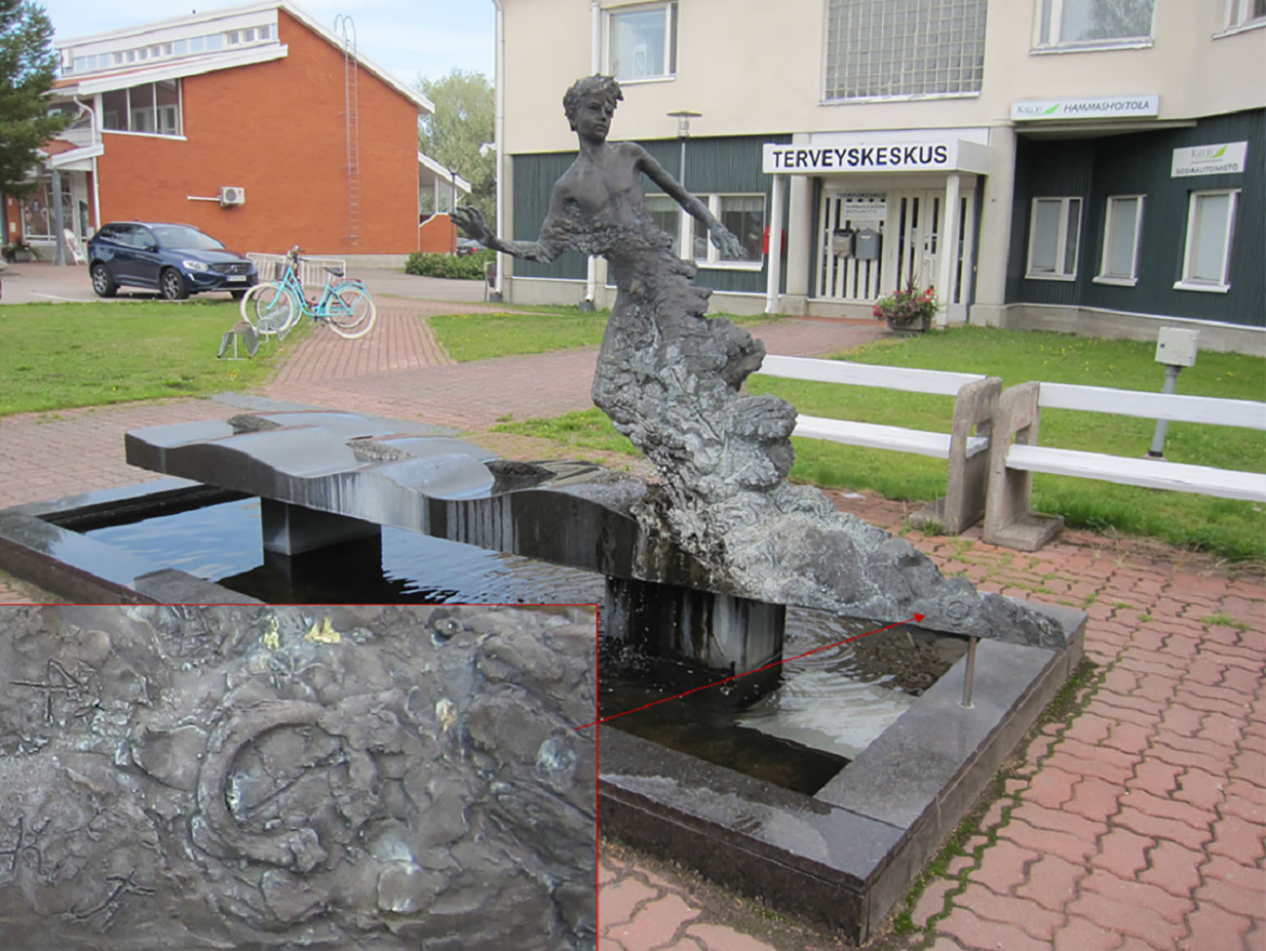
Sculpture *Mutti Matti* or *Sievi Boy* by Taru Mäntynen in front of the municipality hall of Sievi. Photograph: the author

It was the metal artefacts that created most interest in the local public. The media was more discreet in stating the interest (Yle News, 2019). Prior to hobbyists’ and excavation finds, there had been only one known Iron Age metal find from the region, a bronze brooch from the 11th century (see Figure 4) found already in 1911. On the other hand, local people had continuously found Stone Age implements while working in the forest and fields. (See Hakamäki, 2020; Modarress Julin, 2021; Okkonen, 1993.) The general idea of thousands of years of human habitation in the site was also a matter which people found very intriguing.

On the excavations of August 2020, the circumstances had changed quite a lot due to the Covid 19 pandemic. However, since the health situation was a bit better in the summer months and since we were working outdoors, field-classes were held with a small group of archaeology students. The lectures for the wider public were cancelled and visits were not encouraged. Occasionally a few volunteers could take part in the digging, and there were some visits by highly interested locals.^9^

## Methodology and Data

**Figures 5 and 6:**
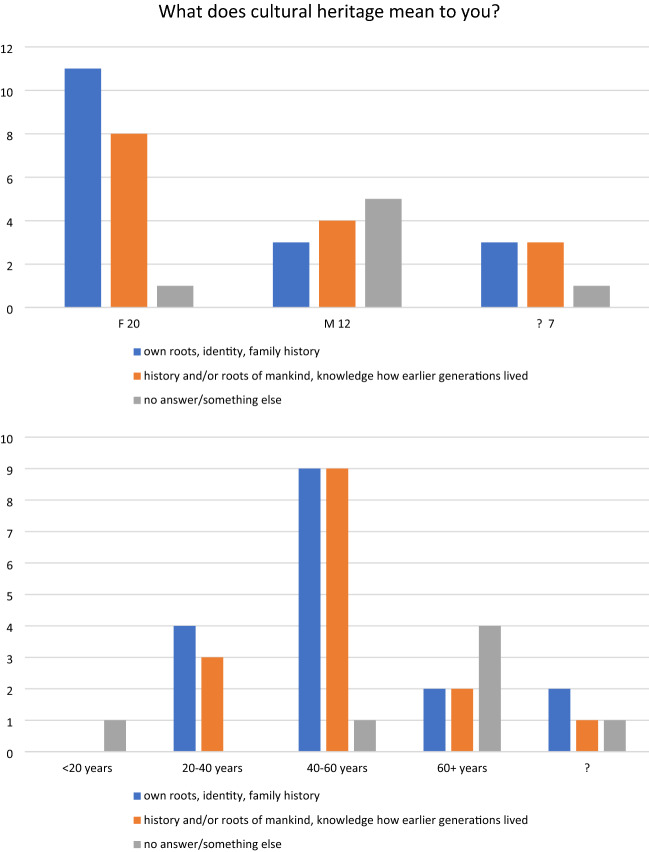
Responses to a brief questionnaire in Sievi 2019 (summary and transl. by the author). There were altogether 39 responses. Up divided according to sex (20 women, 12 men and 7 who did not mention the sex) and down by age-groups: 1 (< 20), 7 (20–40), 19 (40–60), 8 (60+), 4 (?). Only one respondent was under 20 years and four persons did not give their age

To get some idea of the participants' views on cultural heritage I had done a short questionnaire survey (Figures 5, 6 and Table 1) already in the Muttimarkkinat fair, and mainly before the lectures on excavations.^10^ The aim of the inquiry was to acquire a perception of local views, the grassroots view, on archaeology and cultural heritage, to be a platform for later discussions with them.^11^ However, it was hard to get participants to answer official looking forms, and less than half of them responded. People were asked to give short and spontaneous answers, to express their own view and immediate thoughts, and not to look for “right” answers on the Internet in their cell phones. Seemingly, it is not an easy thing to write in a few words one’s views on the paper. I did encourage people to fill in the forms, with scanty results, and one of them remarked: “It’s a hard task, I feel like being back to school.” All in all, there were 39 completed forms. Obviously, the people who came to listen to the lectures were more interested and motivated about the heritage issues than those who took part in the survey in the market-day.

**Table 1:** What is cultural heritage/what does it mean to you?.

N	Sex	Age	Answers
1	F	< 20	What kind of world and life is left to the future generation. I would want to leave a worthy life & world for them
2	F	20–40	Old things, objects, and buildings, perhaps also landscapes. Enables us to understand present and the world when we see how the life was earlier
22	M	20–40	Material and immaterial history and art. Part of identity
15	F	40–60	(CH) unifies people and nation. It is a specific way to think and act. It means belonging to some community/group, it is commitment
37	F	40–60	History, I am interested in it when it’s about my near surroundings or my own roots
23	M	40–60	Heritage, which the people who lived before left for us. It's important that we can "relive" things, objects, and life, which people have lived before us
35	M	40–60	Knowing and preserving one's own country/nation, and I value it a lot
32	U	40–60	Material and immaterial capital, produced by earlier generations and societies. I value the layers of shared material and immaterial heritage of my own culture
36	F	60 +	Cherishing and valuing the past. Reaching for my own roots
39	M	60+	Old things. Does not mean much
26	U	60+	Immaterial and material heritage from the earlier generations. It’s important so that we can understand the present, to see how the things have developed and changed

Answers given by public participants in a brief questionnaire in Sievi (transl. by author)

Almost all (36/39) respondents considered heritage and archaeology meaningful and important. For many of them, cultural heritage was primarily about their own roots, identity, and family history (Figures 5, 6). Women (11 of 19 responses) expressed it as such more often than men (3 of 10) and the others (3 of 6), who considered cultural heritage in the more general terms of legacy and history of past generations. Both materiality and immateriality of heritage were mentioned regularly. Some of the answers are presented in respondents’ own words (Table 1). The locality, the nearness, knowledge of one’s own family history seem to add to the inner meaning of one’s heritage conception. The national aspect and/or global heritage were seemingly more distant matters since they were specifically mentioned only twice. In the nationwide Cultural Heritage Barometer (see Kulttuuriperintöbarometri, 2021, pp. 17, 18, Figure 8a, b) a fair number (61%) of respondents saw cultural heritage affecting their local identity and over half (54%) their own identity, but most respondents (79%) considered it as essential part of Finnish identity.

Often, the interest in heritage and the past seems to increase with age (also e.g., Oniszczuk, 2021, p. 88; Shakour et al., 2019, p. 385). This can be observed also in the Finnish Cultural Heritage barometer 2021 (see Kulttuuriperintöbarometri, 2021, p. 19, Figure 10). When asked how important is to conserve/preserve heritage, from respondents under 30 years 75% answered it is ‘very or rather important’ and the amount increased steadily with the age until in the age-group over 65 years it was 96%. In Sievi, both in survey and excavations, the most active age group to show interest in heritage issues seemed to be over thirty to about sixty years.

Survey participants were also asked a few questions about the hobby of metal detecting, since the research got started from the hobbyists’ finds. There were only three persons (3/39) who said they do metal detecting and one person had tried it, while one was planning to start the hobby. Seemingly, the metal detectorists did not take part in the lectures or fill in the survey form.

## Positioning Heritage in Local Context

My experience from the excavations in Sievi indicated that local people are genuinely interested in archaeology and the past, and not necessarily via metal detecting. The interest in participating in the research or to come to the lectures and/or even just to see the excavations process was favored. Furthermore, as Aalto (2020, p. 161; see also Van Den Dries 2021, p. 215) states, the motive for public participation often includes the desire to learn, so teaching is an essential part of public archaeology.

In Sievi both in the responses to questionnaire and casual discussions “roots” and “ancestors” were words which came up repeatedly. Through the excavation archaeology and history of the home region gets deeper meaning and becomes a matter of collective memory to share with other members of the community. Adults were expecting finds that would more distinctly ‘tell’ something of the past, especially from the past of their home region. One volunteer asked me “while/when you (archaeologists) are excavating do you see the ancient people in your mind’s eye: their dresses, their activities?” There is goal but not much time for contemplation, since excavations are often under-resourced—lack of time, personnel and budged. Formation of the idea how life might have been in the researched period comes later, when the finds are analyzed and interpreted.

For the schoolchildren, in our excavations (both 2019 and 2022), the excitement of finding something from the past was what mattered. They were asking questions such as: ‘Do you find skeletons here?’ or ‘Where did the past people live, I would like to find their house.’ Though, when excavating themselves (2022) even the minor finds like flakes of quarts got them happy, the finder was proudly showing the find to her/his classmates. In the case of schoolchildren, to raise their awareness of the past is giving the seeds to grow the knowledge of the past and to develop an understanding of both personal and collective local identity. Which is the base for understanding also the common past and heritage.

## Materiality, Heritage, and People

Over a decade ago Smith (2006) presented her influential views on intangibility of heritage and heritage as process, views that certainly left their mark on the definition of concept of heritage and influenced subsequent discussions in archaeological literature. Later, Waterton and Smith (2009) also questioned archaeology’s part in the heritage. Nevertheless, I see archaeology to be an essential part of heritage. Sites and artefacts are tangible and create the basis for the intangible heritage process. Archaeology is part of the heritage process, as Carman (2009, pp. 204–206) has expressed by creating different kinds of values which are attached to material objects. Thus, archaeology and heritage go hand in hand (see also Modarress 2015; Modarress-Sadeghi, 2018, pp. 60–62). Heritage is among other things about archaeology, and archaeology is certainly about heritage. According to Meskell (2004, p. 11) “Materiality is our physical engagement with the world, and our way of constituting and shaping culture in an embodied and external sense.” This materiality, I think, is also intimately connected with cultural heritage, and archaeology is studying the material in heritage process. The need for something concrete, material for the people when connecting to heritage was evident in a quite simple way at the excavations in Sievi. Some of the participants when leaving the site, took a small stone with them. They wanted to have something concrete to remind them of the place and the process of excavating the past. The idea is similar when visitors buy a replica of an ancient object or souvenir at a (touristic) heritage site. The replica or the stone is not heritage, but it is a material reminder of the heritage site.

One of the important questions in accessible heritage policy is the use and exploitation of archaeological heritage. Should the utilization of archaeological sites and objects be restricted in some ways, and should we preserve them for future generations? Preserving them does not necessarily mean considering them as unalterable or “frozen” (see Holtorf, 2008), instead it means giving them attention and taking care of them. They can still be acclaimed and used by people in various meaningful ways (Modarress, 2015). Archaeological heritage is generally understood to be collective, something to be cherished by everyone. A heritage process, in which people can take part, define, use, and enjoy the heritage of their ancestors, is an ideal to be strived for (see Faro, 2005). How to reach such an ideal is debated constantly: How heritage would be accessible for all, how to engage various interested parties in the process of learning and defining the past (e.g., Carman, 2005; Lagerlöf 2012; Sayej et al., 2015; Smith, 2006; amongst others). Nevertheless, the decisions and protection are generally left to the heritage officials, and the need of professionals is stated also in the European Convention on the protection of the Archaeological Heritage, Valletta 1992 (Council of Europe, 2001, p. 96). If free to exploit, many a site and countless objects would disappear in the process. Thus, there is a need for maintenance of ancient heritage by the authorities,^12^possibly including some restrictions on their use, even though some hobbyists and researchers might consider that too protective or dictated attitude. When preserving archaeological heritage, there comes inevitable questions as what to preserve and on what grounds the selection is made. The other big question is who decides about it, since preserving everything from the past is impossible and will be more so in the future, as Holtorf (2008, p. 217) also states. The question of what is studied archaeologically is connected to the same dilemma.

There have been well grounded discussions on Authorized Heritage Discourse as presented by Smith (2006) and on the continued critical debate about the officials being in too tight control of heritage. However, some of these discussions do not pay attention to the misconduct from the side of “the public” (see Karl, 2020). Besides the irresponsible metal-detecting, there are wide variety of misuse of archaeological heritage by various groups, like illegal digging and trade, deliberate destruction for land use and building, political destruction in conflicts. The shared archaeological heritage should not be a means in no-one’s self-interest. That is why continuous evaluations and discussions are necessary to keep both authorities and people at large in check. These questions have widely been tackled by archaeologists at least for a couple of decades, but not so much in Finnish archaeology.

## Metal Detecting a Way to Participate in the Heritage Process (?)

Hobby-based metal detecting, when directed to finding ancient artefacts is one way to get involved with the past. Nevertheless, it can be problematic from the viewpoint of shared collective heritage (Modarress & Hakamäki, 2019), since a small group of people affect the common heritage. In Finland, metal detecting as a hobby has grown in popularity during the past few years, and it has become a hot debate also in the archaeological community (see e.g., Häkälä & Sorvali, 2017; Immonen & Kinnunen, 2017, 2020; Knuutinen, 2017; Maaranen, 2016; Modarress & Hakamäki, 2019; Moilanen, 2015; Wessman et al., 2016; amongst others).

Motives for detecting vary. There are metal detectorists for whom the past and local history may be the main concern, for some of them the motive is personal pleasure and excitement of the search. Then again, for some detectorists the main motive is a strive to “higher” personal status and visibility, to become known in one’s local community and detector community, to attain cultural and social capital (see Bourdieu, 1986). In part of cases, the interest in ancient objects can be linked to a self-serving consumer society, where “the self” is the priority not the interest in the past remains as such (see Baudrillard, 1968/2005, p. 89). For some, the possibility of economical profits from the finds can fuel the search. Illegal trade and uncontrolled excavations have been considered a minor problem in Finland (Immonen & Kinnunen, 2020, p. 327; Wessman et al., 2016, p. 87), though they are a big problem on a global scale, regions like the Middle-East, South America and, also many places in Europe (e.g., Brodie et al., 2006, 2022; Gill, 2020; Hardy, 2016; Interpol, 2020; see also Modarress-Sadeghi, 2017).^13^ Different countries have different solutions, rules and practices to deal with this question (see e.g., Barkin 2013; Modarress & Hakamäki, 2019; Open Archaeology, 2016; Ulst, 2013). Finland has been permissive towards metal detectorists and the Heritage law is lax compared to some other European countries such as Sweden, Estonia, or France. In fact, Finland is following in the trails of Denmark and the United Kingdom (see Modarress & Hakamäki, 2019; Wessman et al., 2019).

Although, hobby-based digging with the help of metal detectors is a destructive action for the archaeological record and heritage, conducting excavations is likewise destructive. From the excavations there remains the documentation, the gained information, the conserved and preserved ancient artefacts, even though most of them would go in storage. Whereas from metal detectorists there remains usually a hole in the ground and archaeological context thrown into disarray (e.g., Modarress et al., 2021; see also Maaranen, 2021) and objects either hidden in private collections, traded, or deteriorating in museum stores. Nevertheless, much has been written about responsible detecting as well as its negative sides (see Open Archaeology, 2016, for and against). Undeniably, found objects are sometimes spectacular, and in Finland supposedly rather often given or reported to museums or recorded in common databases. In Finland, the Heritage Agency has an *Ilppari* Internet based reporting site for those who find ancient objects and want to inform about them (www.Museovirasto.fi). According to Raninen (2023) from The Finnish Heritage Agency the number of reported finds has grown during past three years being a bit under 5000 finds last year. Most of them were metal finds. Recently, another Internet-based database called *LöytöSampo* (www.loytosampo.fi/), was launched (see Wessman & Oksanen 2022; Wessman et al., 2019). So far one can find slightly over 3000 recorded though not necessarily verified finds on it. Their descriptions vary greatly. The ancient metal finds and the reported information by detectorists has grown notably also in Northern Ostrobothnia in the past few years,^14^ but the information is based on sporadic finds and not on researched knowledge. As a result of the immense growth (in Finnish scale) of the metal detector finds, the Heritage Agency lacks the resources to check all of them. Likewise, there are not enough resources to conserve the finds, and that is a more serious outcome: the finds deteriorate in their stores (Lähdetluoma, 2021).

The functionality and consequences of the new electronic databases will become apparent in the coming years. For now, discussion is going on to provide people a possibility to take part in heritage process; both to engage them in revealing the material past and at the same time to keep the destruction the hobby creates to archaeological heritage within the limits of sustainability. However, theoretical studies do not always bring forth information about the metal detector offences observed in the field. That might explain the gulf between differences of views between theoretical archaeologists and field archaeologists, who see the actual situation in the field. In theory, it is fine to commend hobbyists “rights to heritage”, but when one works on the field and sees sites disturbed by reckless metal-detecting, it’s a different matter (see e.g., Hänninen, 2021; Kuusela, 2021; Modarress et al., 2021).

One of the aims of the lectures in Sievi was to raise the awareness of the heritage law when using metal detectors in archaeological contexts. In fact, the Finnish Antiquities Act (Fi. *Muinaismuistolaki* 295/1963)^15^ allows only one ancient object-find over one hundred years old to be retrieved. Also, heritage authorities must be informed about the find, and if more objects or a sign of (possible) ancient site are attested, the spot should be left untouched. Nevertheless, dozens of finds have been, and continues to be, carelessly dug up by the hobbyists (Kuusela, 2021; Ruuttula-Vasari, 2021; Tilvis, 2021). One of the consequences of the activity can be that the metal objects are excessively dug, and later field studies will find a mentionable cap of the metal-finds in the archaeological context in Finland (Modarress & Hakamäki, 2019).^16^ Subsequently, that will distort the view of metal periods and the Iron Age would be even harder to discover. From the experience from Sievi, as well as some other places in Finland (e.g., Häkälä & Sorvali, 2017; Knuutinen, 2017; Modarress & Hakamäki, 2019) it has become clear that the metal detector hobbyists, some landowners and others who might come into contact with archaeological sites need to be more aware of the heritage law and sustainable practices concerning ancient sites and objects. Finland has not been very densely populated in ancient times; thus, arguably the Iron Age metal finds deposited underground are a limited nonrenewable resource. Even though there are differences of opinions about the value of ancient heritage being safeguarded and conserved (e.g., Holtorf, 2008), in general it is seen as worthwhile to preserve (see also Kulttuuriperintöbarometri, 2021, p. 19, Figure 10).

## Reinforcing Identity and Heritage

Involvement in heritage can be considered from different viewpoints. There can be conflicting interests of different community groups—locals, officials, researchers, landowners, hobbyists etc., who are connected for instance to economical, recreational, administerial, educational or research needs. When the question is the use and exploitation of the shared archaeological resources, they can and should be considered as we do any other nonrenewable resource (but see e.g., Holtorf, 2008),^17^ and their irresponsible use should not be acceptable (Modarress et al., 2021). Whether the past heritage is appreciated by future generations or not, is not for us to decide. Instead, we should do our best to give them the choice, and not selfishly consume heritage without considering the consequences.

For many Sievi community members the archaeological finds and sites can be a part of roots and identity building (see Figures 5, 6). The discovery of the Iron Age remains was important. Some of them said “at last we move on from Stone Age,” by which they meant progress (since the Stone Age is often understood as primitive in popular views). Whereas, the Iron Age has been mostly considered as a model for “Finnishness” in local contexts as well as national imagery. Iron Age became central during the 19th century when Finland was looking for independence and building up a national identity. It is evident also in archaeology of the period (see Fewster, 1999, 2006). Though views in the archaeology have changed, the old interpretations are sometimes attestable in popular conceptions.

Before Kiurunkangas, in the Sievi region there had been only one known metal-find from the Iron Age, the previously mentioned bronze brooch, which in fact has been memorialized. The meaning of the ancient object as heritage and a source for identity building was realized by Finnish artist Taru Mäntynen in the sculpture “*Mutti Matti*” or “*Sievin poika*” (Sievi Boy) erected in 1998 in the central square in front of the Municipality Hall of Sievi (Figure 4). She depicted the ancient bronze brooch in the sculpture, where it is one of the visible symbols of identity for the community members. For many it is the sign of the (long) age and continuity of the local community. Similarly, the image of the newly found bird-brooch from Kiurunkangas, which is one of the hobbyists’ finds, has been printed on T-shirts as a local symbol. It is obvious that the finds are meaningful for the locals. Furthermore, it is not only about the Iron Age, since the excavation finds represent a long period of human activity of various people coming and going in the region since the Stone Age.

## Conclusions

Meaning(s) of archaeological finds and heritage to the local people is both an enduring and an altering issue. The responses to heritage might change through the years and decades; much depends on the region and locality, but the attraction towards the past remains. In most contexts, the grassroot work with the interested public and their participation in the heritage process and archaeological fieldwork, inviting schoolchildren and youngsters to contemplate the ancient material culture and involving local communities in archaeological fieldwork leads to a better sharing of common heritage on sustainable grounds. Eventually, it increases the understanding our common past.

The Sievi research shows that many locals delight in knowing more about the material signs of their region’s past. To view heritage as an intangible process only is not quite enough (but see Smith, 2006), since material from the past gives heritage physical essence. Thus, archaeology is an essential part of heritage, part of its tangible manifestation. Even a small excavation find can be a meaningful sign of past heritage. Through tangible finds and sites stories and identities are created, without the material component they can remain for many mere vague beliefs or feelings. Taking care, treasuring heritage is considered worthwhile as well as teaching and disseminating knowledge about archaeology and heritage. Taking part in the archaeological excavations or other fieldwork seems to be a pleasing experience for the locals. Many would like to participate in the excavations, even as a minor actor. Those interested in metal detecting could be directed to more sustainable activities like surveying. However, to realize more effective public participation we need the reinforcement of personnel resources of research teams (see also Mikkola, 2010) as well as funding and organizational support. The enduring enthusiasm of the locals and their continuing feedback in Sievi was a rewarding experience and shows that archaeological heritage is a basis for collective identity and above all pleasure invoking matter.

## Footnotes

1 In Finnish archaeology Iron Age is between c.500 BCE to 1200/1300 CE and divided to several sub periods.

2 In 2019 the team consisted of three archaeologists, Jari Okkonen, Ville Hakamäki and the author, accompanied by then a graduate student Emilia Jääskeläinen. The team also included ten novices, students of archaeology completing their compulsory field-school courses. The 2020 team consisted of the author, archaeologist Emilia Jääskeläinen and archaeology student Aleksi Kannussaari together with six other students of Field-school and a few local volunteers. While writing this paper there was one more excavation conducted in Kiurunkangas in the autumn 2022.

3 The list of the research-literature would be too long to refer here, that is why only the used sources are mentioned.

4 The reason is, at least partly, misunderstandings among hobbyists about what archaeologists actually do.

5 While writing this paper the author together with Anne Ruuttula-Vasari got the *Mullankaivajat* grant for the autumn 2022 excavations in Sievi. The excavation is not discussed here because research and the results are not processed and finalized yet. Nevertheless, some remarks and observations from the excavation of 2022 are included.

6 The name is connected to traditional local porrige, *mutti*. It is made from the barley flour, which is poured to boiling water, a bit salt added, and let to boil slowly for many hours. Then it’s eaten hot with a generous chunk of butter.

7 During the last two decades archaeology in Northern Finland has become more active thanks to Oulu University archaeologists.

8 Dr. Anne Ruuttula-Vasari, who has been working for the advancement of the history and heritage knowledge for years in the Sievi community.

9 There was a great interest in excavations, and it was hard to keep the visitors at bay, especially the school-classes would have wanted to increase the visits.

10 Fortunately, I did give lectures (for live audiences) in Sievi, also in the end of February 2020, just before the Covid 19 pandemic started in Finland. During the lectures, it was possible to converse with the local participants, which gave some of the thoughts/opinions expressed in this research.

11 For quantitative research, a wide scale survey on people’s ideas on Cultural Heritage has been done on a national scale. The results can be found online at *Kulttuuriperintöbarometri* 2021, but only in Finnish and a summary in Swedish.

12 Here I speak of authorities taking the rein, but I do not mean forcing their views on others.

13 There is vast literature on illegal market, see also e-Bay antiquities sale pages.

14 Reported finds are listed in the internet pages of The Heritage Agency https://www.kyppi.fi/

15 The Antiquities Act is presently under renewal.

16 See Hardy 2016 for similar thoughts in eastern European context.

17 I agree with Holtorf (2008, p. 127) that there is no need to dig up all the ancient objects. However, if it is done then I think, they should be preserved. In the case of prehistorical objects, it would be crucially important.
